# Patients’ satisfaction with cancer pain treatment at adult oncologic centers in Northern Ethiopia; a multi-center cross-sectional study

**DOI:** 10.1186/s12885-024-12359-7

**Published:** 2024-05-27

**Authors:** Molla Amsalu, Henos Enyew Ashagrie, Amare Belete Getahun, Yophtahe Woldegerima Berhe

**Affiliations:** 1 Department of Anesthesia, Debre Birhan University, Debre Birhan, Ethiopia; 2 Department of Anaesthesia, University of Gondar, Gondar, Ethiopia

**Keywords:** Cancer pain treatment, Patient satisfaction, Treatment satisfaction, Cancer pain, Pain

## Abstract

**Background:**

Patient satisfaction is an important indicator of the quality of healthcare. Pain is one of the most common symptoms among cancer patients that needs optimal treatment; rather, it compromises the quality of life of patients.

**Objective:**

To assess the levels and associated factors of satisfaction with cancer pain treatment among adult patients at cancer centers found in Northern Ethiopia in 2023.

**Methods:**

After obtaining ethical approval, a multi-center cross-sectional study was conducted at four cancer care centers in northern Ethiopia. The data were collected using an interviewer-administered structured questionnaire that included the Lubeck Medication Satisfaction Questionnaire (LMSQ). The severity of pain was assessed by a numerical rating scale from 0 to 10 with a pain score of 0 = no pain, 1–3 = mild pain, 4–6 = moderate pain, and 7–10 = severe pain Binary logistic regression analysis was employed, and the strength of association was described in an adjusted odds ratio with a 95% confidence interval.

**Result:**

A total of 397 cancer patients participated in this study, with a response rate of 98.3%. We found that 70.3% of patients were satisfied with their cancer pain treatment. Being married (AOR = 5.6, CI = 2.6–12, *P* < 0.001) and being single (never married) (AOR = 3.5, CI = 1.3–9.7, *P* = 0.017) as compared to divorced, receiving adequate pain management (AOR = 2.4, CI = 1.1–5.3, *P* = 0.03) as compared to those who didn’t receive it, and having lower pain severity (AOR = 2.6, CI = 1.5–4.8, *P* < 0.001) as compared to those who had higher level of pain severity were found to be associated with satisfaction with cancer pain treatment.

**Conclusion:**

The majority of cancer patients were satisfied with cancer pain treatment. Being married, being single (never married), lower pain severity, and receiving adequate pain management were found to be associated with satisfaction with cancer pain treatment. It would be better to enhance the use of multimodal analgesia in combination with strong opioids to ensure adequate pain management and lower pain severity scores.

## Introduction

Pain is defined as an unpleasant sensory and emotional experience associated with, or resembling that associated with, actual or potential tissue damage [1]. The prevalence of pain in cancer patients is 44.5-66%. with the prevalence of moderate to severe pain ranging from 30 to 38%, and it can persist in 5-10% of cancer survivors [2]. Using the World Health Organization’s (WHO) cancer pain management guidelines can effectively reduce cancer-related pain in 70-90% of patients [3, 4]. Compared to traditional pain states, the mechanism of cancer-related pain is less understood; however, cancer-specific mechanisms, inflammatory, and neuropathic processes have been identified [5]. Uncontrolled pain can negatively affect patients’ daily lives, emotional health, social relationships, and adherence to cancer treatment [6]. Patients with moderate to severe pain have a higher fatigue score, a loss of appetite, and financial difficulties [7]. Patients fear the pain caused by cancer more than dying from the disease since pain affects their physical and mental aspects of life [8]. A meta-analysis of 30 studies stated that pain was found to be a significant prognostic factor for short-term survival in cancer patients [9]. Many cancer patients have a very poor prognosis. However, adequate pain treatment prevents suffering and improves their quality of life. Although the WHO suggested non-opioids for mild pain, weak opioids for moderate pain, and strong opioids for severe pain, pain treatment is not yet adequate in one-third of cancer patients [10].

Patient satisfaction with pain management is a valuable measure of treatment effectiveness and outcome. It could be used to evaluate the quality of care [11–13]. Patient satisfaction affects treatment compliance and adherence [12]. Studies have reported that 60-76% of patients were satisfied with pain treatment, and a variety of factors were found associated with levels of satisfaction [3, 14–16]. Studies conducted in Ethiopia reported the prevalence of pain ranging from 59.9 to 93.4% [17, 18]. These studies indicate that cancer pain is inadequately treated. Assessment of pain treatment satisfaction can help identify appropriate treatment modalities and further its effectiveness. We conducted this study since there was limited research-based evidence on cancer pain management in low-income countries like Ethiopia. Our research questions were: how satisfied are adult cancer patients with pain treatment, and what are the factors associated with the satisfaction of adult cancer patients with pain treatment?

## Methodology

### Study design, area, period, and population

A multi-center cross-sectional study was conducted at four cancer care centers in Amhara National Regional State, Northern Ethiopia from March to May 2023. Those cancer care centers were found in the University of Gondar Comprehensive Specialized Hospital (UoGCSH), Felege-Hiwot Comprehensive Specialized Hospital (FHCSH), Tibebe-Ghion Comprehensive Specialised Hospital (TGCSH) and Dessie Comprehensive Specialized Hospital (DCSH). We selected these centers as they were the only institutions providing oncologic care in the region during the study period.

The UoGCSH had 28 beds in its adult oncology ward and serves 450 cancer patients every month. Three specialist oncologists and 12 nurses provide services in the ward. The FHCSH had 22 beds and provides services for 325 cancer patients every month. Two specialist oncologists, two oncologic nurses, and 7 comprehensive nurses provide services. The TGCSH had eight beds and serves 300 cancer patients every month. There were three specialist oncologists and four oncologic nurses at the care center. The cancer care center at DCSH had 10 beds. It serves 350 cancer patients every month. There was one specialist oncologist, three oncologic nurses, and three comprehensive nurses.

All cancer patients who attended those cancer care centers were the source population, and adult (18+) cancer patients who were prescribed pain treatment for a minimum of one month were the study population. Unconscious patients, patients with psychiatric problems, patients with advanced cancer who were unable to cooperate, and patients with oncologic emergencies were excluded from this study.

### Variables and operational definitions

The outcome variable was patient satisfaction with cancer pain treatment, which was measured by the Lubeck Medication Satisfaction Questionnaire. The independent variables were sociodemographic (age, sex, marital status, monthly income, and level of education), clinical (site of tumor, stage of cancer, metastasis), cancer treatment (surgery, chemotherapy, radiotherapy), level of pain, and analgesia (type of analgesia, severity of pain, adequacy of pain treatment, adjuvant analgesic).

#### Patient satisfaction

perceptions of the patients regarding the outcome of pain management and the extent to which it meets their needs and expectations. It was measured by a 4-point Likert scale (1 = strongly disagree, 2 = disagree, 3 = agree, 4 = strongly agree) using the LMSQ which has 18 items within 6 subscales that have 3 items in each (effectivity, practicality, side-effects, daily life, healthcare providers, and overall satisfaction) [19]. Final categorization was done by dichotomizing into satisfied and dissatisfied by using the demarcation threshold formula.

$(\frac{\text{T}\text{o}\text{t}\text{a}\text{l}\,\,\text{h}\text{i}\text{g}\text{h}\text{e}\text{s}\text{t}\,\,\text{s}\text{c}\text{o}\text{r}\text{e} - \text{T}\text{o}\text{t}\text{a}\text{l}\,\, \text{l}\text{o}\text{w}\text{e}\text{s}\text{t}\,\, \text{s}\text{c}\text{o}\text{r}\text{e} }{2}$) + Total lowest score [20]. The highest patient satisfaction score was 70 and the lowest satisfaction score was 26. A score < 48 was classified as dissatisfied, and a score ≥ 48 was classified as satisfied.

**The Numeric rating scale (NRS)** is a validated pain intensity assessment tool that helps to give patients a subjective feeling of pain with a numerical value between 0 and 10, in which 0 = no pain, 1–3 = mild pain, 4–6 = moderate pain, 7–10 = severe pain [21].

**The Adequacy of cancer pain treatment** was measured by calculating the Pain Management Index (PMI) according to the recommendations of the WHO pain management guideline [22]. The PMI was calculated by considering the prescribed most potent analgesic agent and the worst pain reported in the last 24 h [23]. The prescribed analgesics were scored as follows: 0 = no analgesia, 1 = non-opioid analgesia, 2 = weak opioids, and 3 = strong opioids. The PMI was calculated by subtracting the reported NRS value from the type of most potent analgesics administered. The calculated values of PMI ranged from − 3 (no analgesia therapy for a patient with severe pain) to + 3 (strong opioid for a patient with no pain). Patients with a positive PMI value were considered to be receiving adequate analgesia, whereas those with a negative PMI value were considered to be receiving inadequate analgesia.

### Sample size determination and sampling technique

A single population proportion formula was used to determine the sample size by considering 50% satisfaction with cancer pain treatment and a 5% margin of error at a 95% confidence interval (CI). A non-probability (consecutive) sampling technique was employed to attain a sample size within two months of data collection period. After adjusting the proportional allocation for each center and adding 5% none response, a total of 404 study participants were included in the study: 128 from the University of Gondar Comprehensive Specialized Hospital, 99 from Dessie Comprehensive Specialized Hospital, 92 from Felege Hiwot Comprehensive Specialized Hospital, and 85 from Tibebe Ghion Comprehensive Specialized Hospital.

### Data collection, processing, and analysis

#### Ethical approval

was obtained from the Ethical Review Committee of the School of Medicine at the University of Gondar (**Reference number: CMHS/SM/06/01/4097/2015)**. Data were collected using an interviewer-administered structured questionnaire and chart review during outpatient and inpatient hospital visits by four trained data collectors (one for every center). Written informed consent was obtained from each participant after detailed explanations about the study. Informed consent with a fingerprint signature was obtained from patients who could not read or write after detailed explanations by the data collectors as approved by the Ethical Review Committee of the School of Medicine, at the University of Gondar.

Questions to assess the severity of pain and pain relief were taken from the American Pain Society patient outcome questionnaire [24]. Patients were asked to report the worst and least pain in the past 24 h and the current pain by using a numeric rating scale from 0 to 10, with a pain score of 0 = no pain, 1–3 = mild pain, 4–6 = moderate pain, 7–10 = severe pain.

The Pain Management Index (PMI) based on WHO guidelines, was used to quantify pain management by measuring the adequacy of cancer pain treatment [25]. The following scores were given (0 = no analgesia, 1 = non-opioid analgesia, 2 = weak opioid 3 = strong opioid). Pain Management Index was calculated by subtracting self-reported pain level from the type of analgesia administered and ranges from − 3 (no analgesic therapy for a patient with severe pain) to + 3 (strong opioid for a patient with no pain). The level of pain was defined as 0 with no pain, 1 for mild pain, 2 for moderate pain, and 3 for severe pain. Patients with negative PMI scores received inadequate analgesia.

The pain treatment satisfaction was measured by the Lübeck Medication Satisfaction Questionnaire (LMSQ) consisting of 18 items [19]. Lübeck Medication Satisfaction Questionnaire (LMSQ) has six subclasses each consisting of equally waited and similar context of three items. The subclass includes satisfaction with the effectiveness of pain medication, satisfaction with the practicality or form of pain medication, satisfaction with the side effect profile of pain medication, satisfaction with daily life after receiving pain treatment, satisfaction with healthcare providers, and overall satisfaction. Satisfaction was expressed by a four-point Likert scale (4 = Strongly Agree, 3 = Agree, 2 = Disagree, 1 = Strongly Disagree). The side effect subclass was phrased negatively, marked with Asterix, and reverse-scored in STATA before data analysis.

Data were collected with an interviewer-administered questionnaire. The reliability of the questionnaire was assessed by using 40 pretested participants and the reliability coefficient (Cronbach’s alpha value) of the questionnaire was 91.2%. The collected data was checked for completeness, accuracy, and clarity by the investigators. The cleaned and coded data were entered in Epi-data software version 4.6 and exported to STATA version 17. The Shapiro-Wilk test, variance inflation factor, and Hosmer-Lemeshow test were used to assess distribution, multicollinearity, and model fitness, respectively. Descriptive, Chi-square and binary logistic regression analyses were performed to investigate the associations between the independent and dependent variables. The independent variables with a p-value < 0.2 in the bivariable binary logistic regression were fitted to the final multivariable binary logistic regression analysis. Variables with p-value < 0.05 in the final analysis were considered to have a statistically significant association. The strength of associations was described in adjusted odds ratio (AOR) at a 95% confidence interval.

## Results

### Sociodemographic and clinical characteristics

A total of 397 patients were involved in this study (response rate of 98.3%). Of the participants, 224 (56.4%) were female, and over half were from rural areas (*n* = 210, 52.9%). The median (IQR) age was 48 (38–59) years [Table 1]. The most common type of cancer was gastrointestinal cancer 114 (28.7%). Most of the study participants, 213 (63.7%), were diagnosed with stage II to III cancer. The majority of the participants were taking chemotherapy alone (292 (73.6%)) [Table 2]. Over 90% of patients reported pain; 42.3% reported mild pain, 39.8% reported moderate pain, and 10.1% reported severe pain. Pain treatment adequacy was assessed by self-reports from study participants following pain management guidelines, and 17.1% of patients responded to having inadequate pain treatment. The majority of patients, 132 (33.3%), were prescribed combinations of non-opioid and weak opioid analgesics for cancer pain treatment. Only 34 (8.6%) cancer patients used either strong opioids alone or in combination with non-opioid analgesics.

**Table 1:** Sociodemographic characteristics of adult cancer patients, *n* = 397

Variables	Measurement values, *n* (%)	
**Age**	Median (IQR)	48 (38–59)
**Sex**	Male	173 (43.6)
	Female	224 (56.4)
**Marital status**	Married	305 (76.8)
	Single	39 (9.8)
	Divorced	38 (9.7)
	Widowed	15 (3.8)
**Residency**	Urban	187 (47.1)
	Rural	210 (52.9)
**Level of education**	No formal education	146 (36.8)
	Primary education	125 (31.5)
	Secondary education	79 (19.9)
	College and above	47 (11.8)

IQR: Inter-quartile range

**Table 2:** Clinical characteristics of adult cancer patients, *n* = 397

Variables	Categories	Frequency, *n* (%)
Other systemic illness	Yes	79 (19.9)
	No	318 (80.0)
Types of cancer	Gastrointestinal	114 (28.7)
	Breast	92 (23.2)
	Gynecologic	70 (17.6)
	Lung	26 (6.6)
	Hematologic	29 (7.3)
	Head and neck	20 (5.0)
	Genitourinary	22 (5.5)
	Other cancers	24 (6.0)
Stage of cancer	Stage I	66 (16.2)
	Stage II	147 (37.0)
	Stage III	106 (26.7)
	Stage IV	78 (19.7)
Metastasis	Yes	178 (44.8)
	No	203 (55.2)
Types of cancer treatment	Chemotherapy	292 (73.6)
	Surgery and chemotherapy	91 (22.9)
	Other treatments	14 (3.5)
Types of analgesics	Weak opioid	114 (28.7)
	Weak opioid and non-opioid	132 (33.2)
	Strong opioid and non-opioid	34 (8.6)
	Non-opioids only	117 (29.5)
Pain in the last 24 h	No	31(7.8)
	Mild pain	168 (42.3)
	Moderate to severe	198 (49.9)
Adequacy of pain management	Adequate	329 (82.9)
	Inadequate	68 (17.1)

**Other cancers**: skin, central nervous system, otolaryngologic, orthopedic, pregnancy-related, thymus gland, and cancer of unknown primary origin. **Other treatments**: symptomatic therapy (3), surgery solely (2), radiotherapy (1), chemotherapy and radiotherapy (4), chemotherapy, surgery and radiotherapy (2) symptomatic and chemotherapy (2)

### Patients’ satisfaction with cancer pain treatment and correlation among the subscales

Most participants strongly agree (243, (61.2%)) with item **LMSQ18** in the “overall satisfaction” subscale and strongly disagree (206, (51.9%)) for item **LMSQ2** in the “side-effect” subscale respectively [Table 3]. The highest satisfaction score was observed in the side-effect subscale, with a median (IQR) of 10 (9–11) [Table 4].

**Table 3:** Adult cancer patients’ responses for LMSQ items, *n* = 397

S. No	Items	1, *n* (%)	2, *n* (%)	3, *n* (%)	4, *n* (%)
1.	My medication schedule suits me well	55 (13.8)	72(18.9)	182 (45.8)	85 (21.4)
2. *	I feel restricted in my everyday activities due to the side effects of my medication.	206 (51.9)	135 (34.0)	42 (10.6)	14 (3.5)
3.	My medication is very convenient to take	109 (27.5)	162 (40.8)	100 (25.2)	26 (6.6)
4.	Overall, I am satisfied with my treatment.	38(9.6)	88(22.2)	133(33.5)	138(34.8)
5.	My symptoms are being alleviated by my medication.	43(10.8)	58(14.6)	171(43.1)	125(31.5)
6.	I feel like my physician is educating me properly about my disease.	54(13.6)	67(16.9)	146(36.8)	130(32.8)
7.	I am content with the taste and size of my medications.	39 (9.8)	130(32.8)	177(44.6)	51(12.9)
8.	The advantages and disadvantages of the treatment options were explained to me by my physician in detail.	69 (17.4)	68 (17.1)	141 (35.5)	119 (30.0)
9. *	I am unable to perform as much physical activity as before due to the side effects of my medication.	176 (44.3)	160 (40.3)	45 (11.3)	16 (4.0)
10.	My medication helps me perform personal hygiene tasks (brushing my teeth, taking a shower, etc.).	91 (27.7)	92 (28.3)	158 (27.8)	56 (12.9)
11.	Prior to my treatment, I felt worse than now.	41 (10.3)	55 (13.9)	171 (43.1)	130 (32.8)
12.	The medication helps me get through my everyday life	97 (24.4)	77 (19.4)	184 (43.4)	39 (9.8)
13.	My physician has educated me about the best treatment option	59 (14.9)	80 (20.2)	133 (33.5)	125 (31.5)
14.	I am content with the time passing until my medication starts to work.	94 (23.7)	68 (17.1)	138 (34.8)	97 (24.4)
15.	I am happy with my treatment.	51 (12.9)	79(19.9)	129 (32.5)	138 (34.8)
16.	Thanks to the medication, I can participate in leisure activities	82 (20.7)	82 (20.7)	191(48.1)	42 (10.6)
17. *	I cannot enjoy my leisure time as much anymore due to the side effects of my medication. *	128 (32.2)	183 (46.1)	63 (15.8)	23 (5.8)
18.	I intend to continue my treatment.	10 (2.5)	21 (5.3)	123 (31.0)	243 (61.2)

*: Negatively worded items and reversely coded

1 = I strongly disagree, 2 = I disagree, 3 = I agree, 4 = I strongly agree

**Subscale items LMSQ**: side-effect (2, 9, 17), effectivity (5, 11, 14), practicability (1, 3, 7), daily-life (10, 12, 16), healthcare workers (6, 8,13), overall satisfaction (4, 15, 18)

LMSQ: Lübeck Medication Satisfaction Questionnaire (LMSQ)

**Table 4:** Measurements of LMSQ subscales, *n* = 397

Measurement	Side-effect*	Effectivity	Practicability*	Daily-life	Healthcare worker*	General satisfaction
Mean (SD)	9.6 ± 2.1	8.5 ± 2.7	7.5 ± 2.2	7.1 ± 2.5	8.4 ± 2.8	9.3 ± 2.3
Median (IQR)	10 (9–11)	9 (8–11)	8 (6–9)	8 (6–9)	9 (7–12)	9 (8–12)

*: Non-normally distributed data

LMSQ: Lübeck Medication Satisfaction Questionnaire (LMSQ)

IQR: Inter-quartile Range, SD: Standard Deviation

**Fig. 1:**
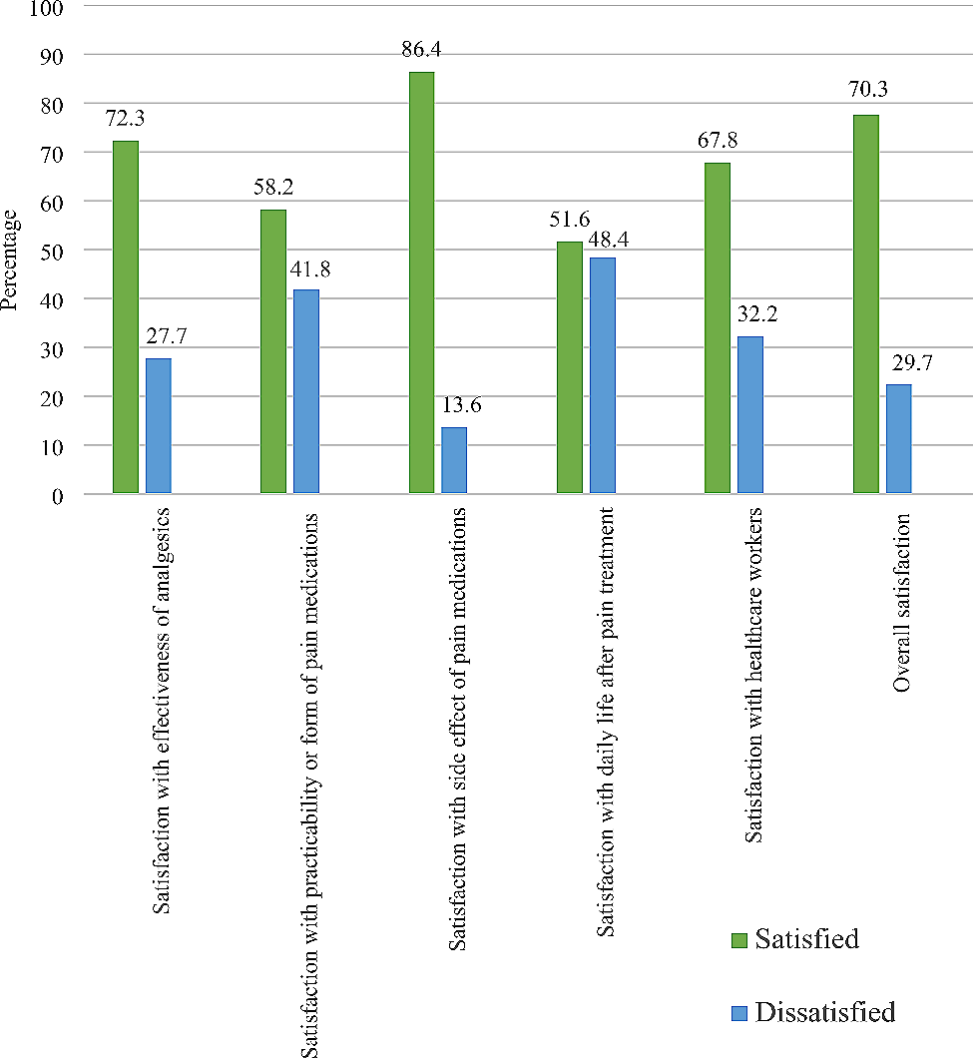
Patient satisfaction with cancer pain treatment with each LMSQ subclass, *n* = 397

**Fig. 2:**
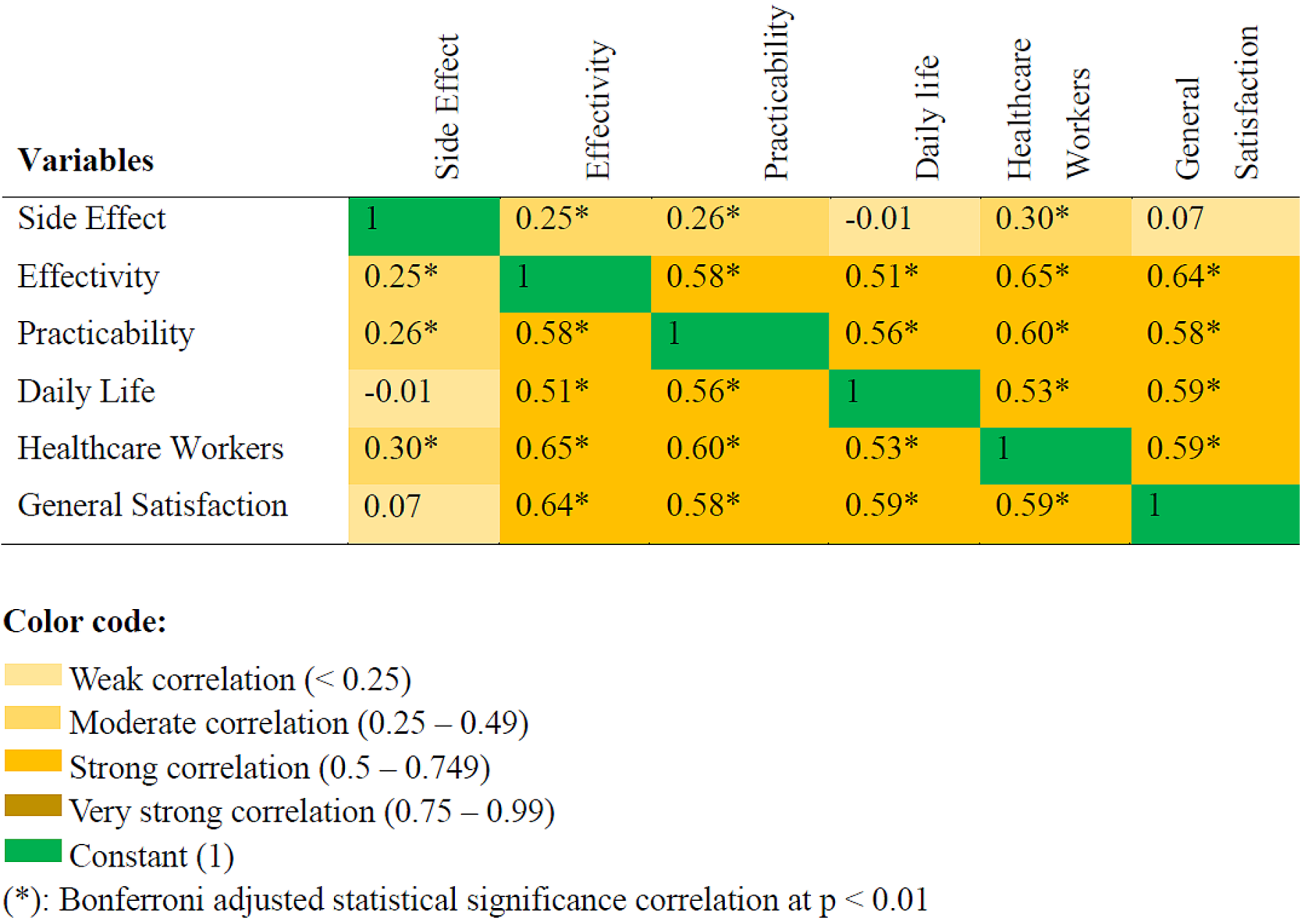
A heatmap showing the Spearman correlation of each subclass of pain treatment satisfaction, *n* = 397

Two hundred and seventy-nine (70.3%) cancer patients were found to be satisfied with cancer pain treatment (CI = 65.6−74.6%). The highest satisfaction rate was observed in the “side-effects” subscale, to which 343 (86.4%) responded satisfied [Fig. 1]. A Spearman’s correlation test revealed that there were correlations among the subscales of LMSQ; and the strongest positive correlation was observed between effectivity and healthcare workers subscale (r_s_ = 0.7, *p* < 0.0001). The correlation among the subscales is illustrated in a heatmap [Fig. 2].

### Factors associated with patient satisfaction with cancer pain treatment

In the bivariable binary logistic regression analysis, marital status, stage of cancer, types of cancer treatment, severity of pain in the last 24 h, current pain severity, types of analgesics, and pain management index met the threshold of P-value < 0.2 to be included into the final multivariable binary logistic regression analysis. In the final analysis, marital status, current pain severity, and pain management index were significantly associated with patient satisfaction (P-value < 0.05). Married and single cancer patients had higher odds of being satisfied with cancer pain treatment compared to divorced patients (AOR = 5.6, CI, 2.6–12.0, *P* < 0.001), (AOR = 3.5, CI = 1.3–9.7, *P* = 0.017), respectively. The odds of being satisfied with cancer pain treatment among patients who received adequate pain management were more than two times greater than those who received inadequate pain management (AOR = 2.4, CI = 1.1–5.3, *P* = 0.03). Patients who reported a lesser severity of current pain were nearly three times more likely to be satisfied with cancer pain treatment (AOR = 2.6, CI = 1.5–4.8, *P* < 0.001) [Table 5].

**Table 5:** Factors associated with patients’ satisfaction with cancer pain treatment; Bivariate and multivariate binary logistic regression, *n* = 397

Variables	Categories	Satisfaction status		Odds ratio (95% CI)		P-Value
		Dissatisfied n (%)	Satisfied n (%)	COR	AOR	
Marital status	Married	75 (24.6)	230 (75.4)	4.2 (2.1–8.4)	5.6 (2.6–12)	**< 0.001**
	Single	14 (35.9)	25 (64.1)	2.5 (0.9–6.1)	3.5 (1.3–9.7)	**0.017**
	Widowed	7 (46.7)	8 (53.3)	1.6 (0.4–5.2)	2.5 (0.6–9.5)	0.19
	Divorced	22 (57.9)	16 (42.1)	1	1	1
Stage of cancer	Stage III and IV	85 (26.6)	234 (73.4)	2 (1.2–3.4)	1.3(0.7–2.4)	0.37
	Stage I and II	33 (42.3)	45 (57.7)	1	1	1
Type of cancer treatment	Chemotherapy and surgery	20 (22)	71 (78)	1.7 (1–2.9)	1.8 (1.0–3.3)	0.07
	Radiotherapy	4 (28.6)	10 (71.4)	1.2 (0.4–3.9)	1.5 (0.8–2.9)	0.54
	Chemotherapy	94 (32.2)	198 (67.8)	1	1	1
Types of analgesics	Weak opioid	37 (32.4)	77 (67.6)	1.3 (0.8–2.3)	1.4 (0.7–2.9)	0.30
	Weak opioid and non-opioid	27 (20.4)	105 (79.5)	2.5 (1.4–4.4)	1.9 (0.9–3.9)	0.07
	Strong opioid and non-opioid	8 (23.5)	26 (76.5)	2.1 (0.9–5)	2.1 (0.7–6.4)	0.18
	Non-opioids only	46 (39.3)	71 (60.7)	1	1	1
Worst pain in last 24 h	No to mild pain	37 (18.6)	162 (81.4)	3 (1.9–4.7)	1.5 (0.8–2.9)	0.20
	Moderate to severe	81 (40.9)	117 (59.1)	1	1	1
Adequacy of pain management	Adequate	42 (61.7)	26 (38.3)	5.4 (3.1–9.3)	2.4 (1.1–5.3)	**0.03**
	Inadequate	76 (23.1)	253 (76.9)	1	1	1
Current pain severity	No to mild pain	65 (22.3)	227 (77.7)	3.6 (2.2–5.7)	2.6 (1.5–4.8)	**< 0.001**
	Moderate to severe	53 (50.5)	52 (49.5)	1	1	1

AOR: Adjusted Odds Ratio, CI: Confidence Interval, COR: Crude Odds Ratio

Bold values: Significant in multivariate binary logistic regression analysis

## Discussion

The objective of the present study was to assess patients’ satisfaction with cancer pain treatment at adult oncologic centers. Our study revealed that most cancer patients (70.3%) have been satisfied with cancer pain treatment. This is consistent with studies done by Kaggwa et al. and Mazzotta et al. [16, 26]. Whereas, it is a higher rate of satisfaction compared to other studies that reported 33.0% [27] and 47.7% [28] of satisfaction. The differences might be possibly explained by the use of different pain and satisfaction assessment tools, the greater inclusion (about 70%) of patients with advanced stages of cancer, the duration of cancer pain treatment, and the adequacy of pain management. In the current study, only 19.6% of patients have been diagnosed with stage IV cancer: patients should take treatment at least for a month, and over 80% of patients have received adequate pain management according to PMI. However, some studies have reported higher rates of satisfaction with cancer pain treatment [15, 29]. The possible reason for the discrepancy might be the greater (over 40%) use of strong opioid analgesics in the previous studies. Strong opioids were prescribed only for 8.6% of patients in our study. Due to the complex pathophysiology, cancer pain involves multiple pain pathways. Hence, multimodal analgesia in combination with strong opioids is vital in cancer pain management [30]. Furthermore, the use of epidural analgesia could be another reason for higher rates of satisfaction [29].

Regarding satisfaction with subscales of LMSQ, about 80% of patients were satisfied with the information provided by the healthcare providers [27]. In our study; 67.8% of patients were satisfied with the education provided by healthcare providers about their disease and treatment. In contrast, a higher proportion of participants were satisfied with information provision in a study conducted by Kharel et al. [29]. Furthermore, we observed the lowest satisfaction rate in the daily life subscale. About 48% of cancer patients were not satisfied with their daily lives after receiving analgesic treatment for cancer pain.

Married and single (never married) cancer patients were found to have higher odds of being satisfied with cancer pain treatment as compared to divorced cancer patients. These findings could be explained by the presence of better social support from family or loved ones. Better social support can enhance positive coping mechanisms, increase a sense of well-being, and decrease anxiety and depression. It also improves a sense of societal vitality and results in higher patient’ satisfaction [31, 32].

Patients who had a lower pain score were satisfied compared to those who reported a higher pain score, and this is supported by multiple previous studies [16, 26, 27, 29, 33, 34]. This could be explained by the negative impacts of pain on physical function, sleep, mood, and wellbeing [35]. Moreover, higher pain severity scores could increase financial expenses because of unnecessary or avoidable emergency department visits; and has a consequence of dissatisfaction [23]. On the contrary, there are studies that state pain severity does not affect patients’ satisfaction [36, 37].

Positive PMI scores were significantly associated with cancer pain treatment satisfaction. Patients who received adequate pain management were highly likely to be satisfied with cancer pain treatment. This finding is similar to that of a study done in Taiwan [38]. However, a study conducted by Kaggwa et al. has denied any association between PMI scores and cancer pain satisfaction [16].

### Satisfaction with healthcare workers and effectivity of analgesics

This study showed that there was a moderately positive correlation between satisfaction with healthcare workers and satisfaction with patients’ perceived effectiveness of analgesics. This might be explained by a positive relationship between healthcare professionals and patients receiving cancer pain treatment. Healthcare providers who provide health education regarding the effectiveness of analgesics may improve patients’ adherence to the prescribed analgesic agent and improve patients’ perceived satisfaction with the effectiveness of analgesics. A systematic review showed that the hope and positivity of healthcare professionals were important for patients to cope with cancer and increase satisfaction with care [39]. Increased patient satisfaction with care provided by healthcare workers may change attitude of patients who accepted cancer pain as God’s wisdom or punishment and create a positive attitude toward the effectiveness of analgesics [40]. Another study supported this finding and stated that healthcare providers who deliver health education regarding the prevention of drug addiction, side effects of analgesics, timing, and dosage of analgesics improve patient attitude and cancer pain treatment [41].

### Correlation of each subclass of cancer pain treatment satisfaction

A Spearman correlation was run to assess the correlation of each subclass of LMSQ using the total sample. There was strong positive correlation (r_s_ = 0.5–0.64) between most of LMSQ subclass at *p* < 0.01.

A cross-sectional study stated that the effectiveness of analgesic, efficacy of medication and patient healthcare provider communication were associated with patient satisfaction [42]. In this study, 58.2% of patients were satisfied with the practicability of analgesic medications. Comparable to this study, a cross-sectional study stated that patients who were prescribed convenient, fast-acting medications were more satisfied [43]. Another study stated that 100% of patients who received sufficient information on analgesic treatment and 97.9% of patients who received sufficient information about the side effects of analgesic treatment were satisfied with cancer pain management [44]. Patients who were satisfied with their pain levels reported statistically lower mean pain scores (2.26 ± 1.70) compared to those not satisfied (4.68 ± 2.07) or not sure (4.21 ± 2.21) [27]. This may be explained by the impact of pain on daily activity. Patients who report a lower average pain score may have a lower impact of pain on physical activity compared to those who report a higher mean pain score. Another study also supports this evidence and states that patients who reported a severe pain score and lower quality of life had lower satisfaction with the treatment received [45].

As a secondary outcome, only 16% of patients were diagnosed to have stage I cancer. This finding could indirectly indicate that there were delays in cancer diagnosis at earlier stage. Further studies may be required to underpin this finding.

In this study, baseline pain before analgesic treatment was not assessed and documented. As a cross-sectional study, we could not draw a cause-and-effect conclusion. Since questions that were used to measure oncologic pain treatment satisfaction were self-reported, answers to each question might not be trustful. The expectation and opinion of the interviewer also might affect the result of the study. These could be potential limitations of the study.

## Conclusions

Despite the fact that most cancer patients reported moderate to severe pain, there was a high rate of satisfaction with cancer pain treatment. It would be better if hospitals, healthcare professionals, and administrators took measures to enhance the use of multimodal analgesia in combination with strong opioids to ensure adequate pain management, lower pain severity scores, and better daily life. We also urge the arrangement of better social support mechanisms for cancer patients, the improvement of information provision, and the deployment of professionals who have trained in pain management discipline at cancer care centres.

## Data Availability

Data and materials used in this study are available and can be presented by the corresponding author upon reasonable request.

## Abbreviations

AOR: Adjusted Odds Ratio
COR: Crude Odds Ratio
CI: Confidence Interval
DCSH: Dessie Compressive and Specialized Hospital
FHCSH: Felege-Hiwot Compressive and Specialized Hospital
IQR: Inter-quartile Range
LMSQ: Lubeck Medication Satisfaction Questionnaire
NRS: Numerical Rating Scale
PMI: Pain Management Index
SD: Standard Deviation
TGCSH: Tibebe-Ghion Compressive and Specialized Hospital
UoGCSH: University of Gondar Compressive and Specialized Hospital
WHO: World health organization
